# Tongxinluo attenuates reperfusion injury in diabetic hearts by angiopoietin-like 4-mediated protection of endothelial barrier integrity via PPAR-α pathway

**DOI:** 10.1371/journal.pone.0198403

**Published:** 2018-06-18

**Authors:** Kang Qi, Xiangdong Li, Yongjian Geng, Hehe Cui, Chen Jin, Peihe Wang, Yue Li, Yuejin Yang

**Affiliations:** 1 State Key Laboratory of Cardiovascular Disease, Fuwai Hospital, National Center for Cardiovascular Disease, Chinese Academy of Medical Sciences and Peking Union Medical College, Beijing, China; 2 Division of Cardiovascular Medicine, University of Texas Health Science Center at Houston, Houston, TX, United States of America; 3 Peking Key Laboratory for Pre-clinical Evaluation of Cardiovascular Implant Material, State Key Laboratory of Cardiovascular Disease, National Center for Cardiovascular Diseases, Animal Experimental Center, Fuwai Hospital, Chinese Academy of Medical Sciences and Peking Union Medical College, Beijing, China; Virginia Commonwealth University Department of Internal Medicine, UNITED STATES

## Abstract

**Objective:**

Endothelial barrier function in the onset and Tongxinluo (TXL) protection of myocardial ischemia/reperfusion (I/R) injury, and TXL can induce the secretion of Angiopoietin-like 4 (Angptl4) in human cardiac microvascular endothelial cells during hypoxia/reoxygenation. We intend to demonstrate whether TXL can attenuate myocardial I/R injury in diabetes, characterized with microvascular endothelial barrier disruption, by induction of Angptl4-mediated protection of endothelial barrier integrity.

**Methods and results:**

I/R injury was created by coronary ligation in ZDF diabetic and non-diabetic control rats. The animals were anesthetized and randomized to sham operation or I/R injury with or without the exposure to insulin, rhAngptl4, TXL, Angptl4 siRNA, and the PPAR-α inhibitor MK886. Tongxinluo, insulin and rhAngptl4 have the similar protective effect on diabetic hearts against I/R injury. In I/R-injured diabetic hearts, TXL treatment remarkably reduced the infarct size, and protected endothelial barrier integrity demonstrated by decreased endothelial cells apoptosis, microvascular permeability, and myocardial hemorrhage, fortified tight junction, and upregulated expression of JAM-A, integrin-α5, and VE-cadherin, and these effects of TXL were as effective as insulin and rhAngptl4. However, Angptl4 knock-down with siRNA interference and inhibition of PPAR-α with MK886 partially diminished these beneficial effects of TXL and rhAngptl4. TXL induced the expression of Angptl4 in I/R-injured diabetic hearts, and was canceled by Angptl4 siRNA and MK886. TXL treatment increased myocardial PPAR-α activity, and was abolished by MK886 but not by Angptl4 siRNA.

**Conclusions:**

TXL protects diabetic hearts against I/R injury by activating Angptl4-mediated restoration of endothelial barrier integrity via the PPAR-α pathway.

## Introduction

Timely reopening of the occluded coronary artery by mechanical or pharmacological intervention is critical for reduction of tissue injury in patients with acute myocardial infarction (AMI). However, it may trigger ischemia/reperfusion (I/R) injury, aggravating myocardial injury, decreasing and even counteracting the benefit of coronary blood flow restoration[1].

Tongxinluo (TXL), a special formula of Chinese traditional medicines, is composed of *Radix ginseng*, *Buthus martensi*, *Hirudo*, *Eupolyphaga seu steleophaga*, *Scolopendra subspinipes*, *Periostracum cicadae*, *Radix paeoniae rubra*, *Semen ziziphi spinosae*, *Lignum dalbergiae odoriferae*, *Lignum santali albi*, and *Borneolum syntheticum*. TXL was registered in the State Food and Drug Administration of China for treatment of angina pectoris in 1996. Recent studies confirmed that as a stable formulation, TXL capsule could stabilize coronary plaque [2], protecting the myocardium from I/R injury, reducing the size of myocardial necrosis and no-reflow, and improving cardiac performance [3–6]. Therefore in China TXL has been extensively used for the treatment of patients with any clinical type of coronary artery disease, including acute coronary syndrome. The mechanism of TXL against myocardial I/R injury involved the activation of protein kinase A (PKA)/endothelial nitric oxide synthase (eNOS) pathway, protection of microvascular endothelial integrity by up-regulating VE-cadherin, β-catenin, and γ-catenin, and subsequent reduction of myocardial hemorrhage, inflammation, oxidization, edema, apoptosis and necrosis, while eNOS inhibitor (Nω-Nitro-L-arginine) L-NNA completely canceled these effects of TXL, indicating that endothelial barrier function plays a key role in the development and TXL protection of myocardial I/R injury [3–6]. However, the exact protector and regulative mechanism of TXL against myocardial I/R injury are far from clear. Recently, we found that TXL can directly reduce the apoptosis of cardiac microvascular endothelial cells by triggering autophagy [7] through modulating cytokines secretion [8], and angiopoietin-like protein 4 (Angptl4) was one of the dramatically up-regulated cytokines [8], indicating Angptl4 may be an important mediator in the cardioprotective effect of TXL against I/R injury.

Angptl4, a member of the angiopoietin-like gene family, is induced under hypoxic condition in various cell types, and is the target of peroxisome proliferator-activated receptors (PPAR). Angptl4 has been shown to reduce the size of myocardial infarction and no-reflow in rats, and this effect is attributed to the preservation of vascular integrity by protecting VE-cadherin complex [9]. Thus, the induction of Angptl4 secretion in response to I/R injury might be part of the mechanism that TXL protecting endothelial cells and myocardial I/R injury. PPAR-α, rich in myocardium, is part of the PPAR subfamily, and is activated under conditions of energy deprivation. It has been reported that, under hypoxia, stress, and hyperglycemia, PPAR-α can activate Angptl4 expression [10,11]. However, it is unknown whether the PPAR-α/Angptl4 pathway plays a role in TXL-mediated effect against myocardial I/R injury.

Our previous studies were all based on I/R model of normal animals. Most of the AMI patients are based on pathological conditions, such as hypertension, hyperlipidemia, and diabetes mellitus. Especially, diabetes is characterized by dysfunction of endothelial cells and disruption of endothelial barrier integrity [12,13]. Therefore, AMI patients with diabetes often have complicated and exacerbated I/R injury [14]. TXL has been confirmed that can protect against diabetic nephropathy, a kind of microvascular disease, by ameliorating renal structure and function in diabetic rats [15]. But it is unclear whether TXL can also protect endothelial barrier integrity and reduce I/R injury in diabetic hearts.

Therefore, the present study was conducted to determine whether TXL protects hearts from I/R injury in diabetic hearts by stimulating Angptl4-mediated restoration of endothelial barrier integrity via the PPAR-α pathway.

## Material and methods

### Animal experiment protocol

104 male 12-week-old Zucker diabetic fatty (ZDF) rats (Lepr^fa/fa^), weighing 325±25g and blood glucose>16.8 mM for 1-month, were purchased from Vital River Laboratory Animal Technology Co. Ltd., Beijing, China. All rats were type 2 diabetic except that in the non-diabetic MI control group. The animal research protocol was approved by the Institutional Animal Welfare Committee of Fuwai Hospital (No. 2013-4/5-100/30-973) and conformed to the ARRIVE and NIH guidelines.

Rats were anesthetized with intraperitoneal injection of pentobarbital sodium (1%, 35 mg/kg), and anesthesia was maintained with additional doses of pentobarbital (1%, 7 mg/kg/h). Each rat was ventilated with ventilator. After hemodynamic stabilization, each group of rats underwent 45min ligation and 3h reperfusion of the left anterior descending coronary artery (LAD). All rats were randomly assigned to 13 groups (n = 8 in each group): (1) Diabetic sham (DB-sham) group: LAD was only encircled by a suture, but not occluded; (2) Diabetic MI control (DB-MI) group: no intervention either before or after LAD occlusion; (3) Non-diabetic MI control (non-DB-MI) group: no intervention either before or after LAD occlusion; (4) Insulin group: Blood glucose level was maintained at about 10 mM with intravenous injection of insulin (5 IU/100g) and additional doses of insulin (1%, 0.1 IU/100g/min). Blood samples were extracted from the rat tail peripheral capillary every 10min before ligation of LAD to detect the levels of blood glucose, with the help of Accu-CHEK Performa blood glucose meter (Roche, Mannheim, Germany); (5) Recombinant human Angptl4 (rhAngptl4) group: rhAngptl4 (10 μg/kg) was intravenously administered 1h before ischemia; (6) TXL group: TXL (50 mg/kg) was intragastrically administered 1h before ischemia; (7) rhAngptl4+control siRNA (rhAngptl4+siCtrl) group: Rats were anesthetized with intraperitoneal injection of pentobarbital sodium (1%, 35 mg/kg), and anesthesia was maintained with additional doses of pentobarbital (1%, 7 mg/kg/h). Each rat was ventilated with ventilator. The heart was exposed via a left lateral thoracotomy at the 4th intercostal space, followed by a pericardiotomy. 10 μl (0.8 μg/μl) of rat-specific control siRNA (Applied Biosystems, UK), mixed with in vivo jet PEI according to the manufacturer’s instruction (Genesee Scientific, USA) was delivered with microliter syringes (HAMILTON,80365,10μl, Switzerland) via five separate intramyocardial injections into the left ventricular free wall 48h before LAD occlusion, and rhAngptl4 was administered 1h before ischemia; (8) TXL+control siRNA (TXL+siCtrl) group: rats were treated with TXL and control siRNA; (9) rhAngptl4+Angptl4 siRNA (rhAngptl4+siR) group: rats were treated with rhAngptl4 and Angptl4 siRNA; (10) TXL+Angptl4 siRNA (TXL+siR) group: rats were treated with TXL and Angptl4 siRNA; (11) rhAngptl4+MK886 group: 1h before LAD occlusion, PPAR-α inhibitor MK886 (0.4 mg/kg, Tocris Bioscience, USA) was intravenously administered in combination with rhAngptl4; (12) TXL+MK886 group: TXL and MK886 were administered 1h before LAD occlusion; and (13) MK886 group: MK886 was administered 1h before LAD occlusion.

### Measurement of myocardial ischemic and necrotic area

Myocardial area at risk (AAR) was determined by Evens blue staining, and the area of necrosis (AN) was detected by triphenyltetrazolium chloride (TTC) staining according to previous report[3]. Briefly, after 3h of reperfusion, LAD was re-ligated, and 3 ml of 2% Evans blue was injected into left atrium. The heart was excised after the rat was sacrificed by injection of 15% potassium chloride (0.5 ml per animal). Then, the atria and right ventricular free wall were removed, and the remaining left ventricular (LV) tissue was sectioned perpendicular to its long axis into six to seven sections. Each tissue slice was weighed and photographed. AAR, the area unstained by Evans blue, was traced and pictured in visible light. Tissue samples were collected from ischemic and non-ischemic area and were immediately placed in liquid nitrogen for further examination. Finally, tissue slices were incubated in 1% of TTC solution (pH 7.4) at 37°C for 15min to identify AN. TTC stains viable myocardium red, and necrotic tissue appears pale. After computerized planimetry, the percentage of the area was multiplied by the slice weight and then accumulated for different myocardial slices. Outcomes were calculated as follows: AAR (%) = (AAR mass/ LV mass)×100%; AN (%) = (AN mass /AAR mass)×100%.

### Measurement of serum CK activity and cTnI level

To evaluate the extent of myocardial injury, serum creatine kinase (CK, Nanjing Jiancheng Bioengineering Institute, China) activity and serum cTnI (Shanghai BlueGene Biotech, China) level were measured at baseline and after 3h of reperfusion according to the manufacturer’s instructions.

### Measurement of blood glucose level

Following a 12-h fast, blood samples were extracted from the rat tail peripheral capillary at baseline and at 180min after reperfusion to detect the levels of blood glucose, with the help of Accu-CHEK Performa blood glucose meter (Roche, Mannheim, Germany).

### Determination of microvascular permeability

Microvascular permeability in the control and I/R-injured myocardium was determined by fluorescein-isothiocyanate (FITC)-dextran (10%; 70 kDa, Sigma, USA) staining as previous described [16]. Briefly, FITC-dextran was administered via tail vein 30min after reperfusion. Tissue samples (100 mg), collected from non-ischemic and ischemic area of the heart, were rinsed, homogenized and cleared by centrifugation. The fluorescent concentration in the supernatant was measured with a fluorescence spectrophotometer via fluorescence at 485 nm excitation/535 nm emission wavelengths.

### Analysis of myocardial hemorrhage

Myocardial paraffin sections of 10 μm thick were stained with hematoxylin-eosin (HE) and examined under light microscopy to evaluate myocardial hemorrhage as previously described [17]. Local hemorrhage was scored by assessing the red blood cell extravasation as follows: 0, no extravasation; 1, slight extravasation; 2, extensive infiltration of the interstitial space; and 3, areas of confluent intramyocardial hematoma. All sections were analyzed in a blinded manner. All pictures are representative of 8 independent experiments. The data were averaged for each animal group.

### Identification of apoptotic endothelial cells by TUNEL staining

To detect the apoptosis of endothelial cells in I/R-injured myocardium, double staining was performed with CD34 and terminal deoxynucleotide transferase-mediated dUTP nick-end labeling (TUNEL) as previously described [18]. Endothelial cells were identified by red fluorescence (CD34), total cell number was detected by blue fluorescence (DAPI DNA staining), and apoptosis was detected by green fluorescence (TUNEL). Apoptotic endothelial cells were detected and counted by colocalized red and green (displayed as yellow) fluorescence at 40 magnification. Quantification of apoptotic endothelial cells was expressed as an average of the ratio of number of apoptotic endothelial cells per square millimeter to total number of endothelial cells (CD34 staining) per square millimeter.

### Observation of tight junction by transmission electron microscopy

Tight junctions were observed by transmission electron microscopy. Briefly, myocardial samples from area at risk (n = 3 in each group) were fixed in 2% gluteraldehyde (0.1 M, pH 7.4) for 2h, washed with PBS for 2h, and post-fixed in 1% OsO4 for 2h. Each piece of heart tissue was divided into 3 segments for histological analysis. Samples were dehydrated through a graded series of ethanol and embedded in epoxy resin. Semithin sections (0.2 μm) were stained with toluidine blue for light microscopy examinations and were used to guide sampling for transmission electron microscopy studies. Thin sections (0.09 μm) were collected on 150-mesh copper grids and double stained with uranyl acetate and lead citrate for electron microscopy examinations (JEM-1010, Japan). Tight junctions were observed on electron microscopy sections that cut perpendicular to the long axis of the vessel wall. All pictures are representative of 3 independent experiments.

### Western blot analysis

Protein was extracted from heart tissue, collected from the area of non-ischemia and I/R injury. 10 μg of protein from each sample was subjected to SDS–polyacrylamide gel electrophoresis and transferred onto a PVDF membrane (Millipore Corp., USA). The membranes were then incubated with the following antibodies: rabbit monoclonal JAM-A (1:1000 dilution, no. ab52647, Abcam, UK), rabbit monoclonal integrin-α5 (1:1000 dilution, ab150361, Abcam, UK), rabbit monoclonal VE-cadherin (1:1000 dilution, sc-28644, Santa-cruz, USA), rabbit monoclonal GAPDH (1:1000 dilution, no. 5174, CST, USA), or rabbit polyclonal Angptl4 (1:1000 dilution, ab196746, Abcam, UK). Bands were visualized using Chemiluminescent HRP Substrate (Millipore Corp., USA). The intensity ratio of objective band to GAPDH corresponded to the relative amounts of objective protein.

### Analysis of PPAR-α activity

Nuclear protein of I/R-injured myocardium was extracted using nuclear extraction reagents (78835, Thermo, USA). Briefly, 100 mg of heart tissue was cut into small pieces and was homogenized on ice in Cytoplasmic Extraction ReagentⅠ(CERⅠ, 1 ml/100 mg tissue). After fully suspended the pellet, the homogenate was incubated on ice for 10min and ice-cold CERⅡ was added. PPAR-α activity was measured using PPAR-α Transcription Factor Assay kit according to manufacturer’s protocol (ab133107, Abcam, UK).

### Statistics

The data are expressed as mean ± SD. Statistical analysis was performed with one-way ANOVA followed by Tukey’s test (SPSS 20.0). Differences were considered to be significant at *P*<0.05.

## Results

### TXL treatment reduces infarction in the diabetic hearts undergoing I/R injury independent of the reduction of blood glucose

**Fig 1:**
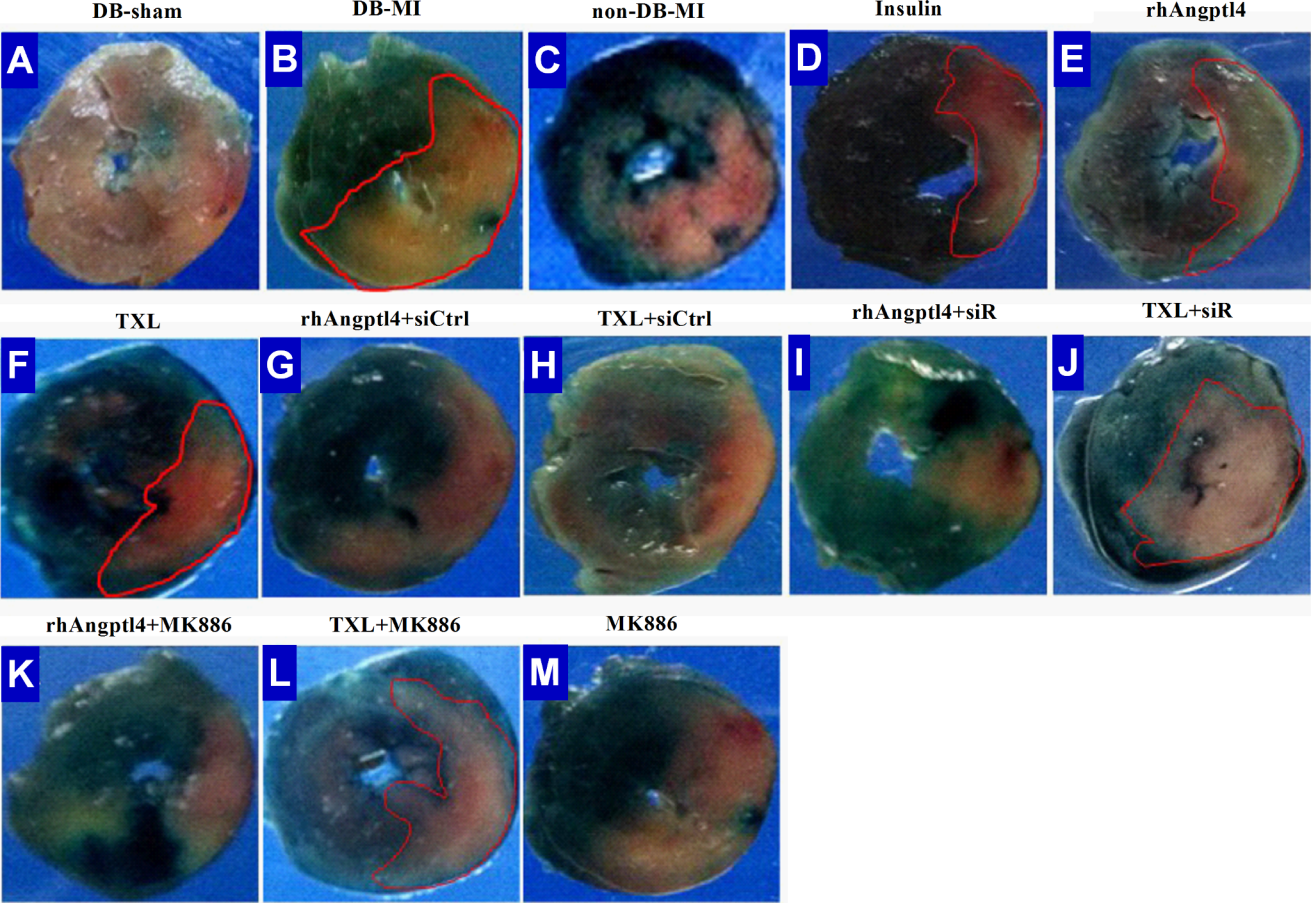
Histopathologic assessments of the area at risk and necrosis in the infarcted hearts treated with or without TXL in the presence or absence of signal regulators. The area at risk and necrosis was respectively examined by Evans blue and triphenyltetrazolium chloride (TTC) staining (n = 8 in each group). The health myocardium was stained blue by Evans blue, the area at risk (AAR) was not stained by Evans blue. TTC-unstained white myocardium was identified as the area of necrosis (AN). Abbreviations: DB-sham = Diabetic sham; DB-MI = Diabetic MI control; non-DB-MI = non-diabetic MI control; rhAngptl4 = recombinant human Angptl4; rhAngptl4+siCtrl = rhAngptl4+control siRNA; TXL+siCtrl = TXL+control siRNA; rhAngptl4+siR = rhAngptl4+Angptl4 siRNA; TXL+siR = TXL+Angptl4 siRNA.

**Fig 2:**
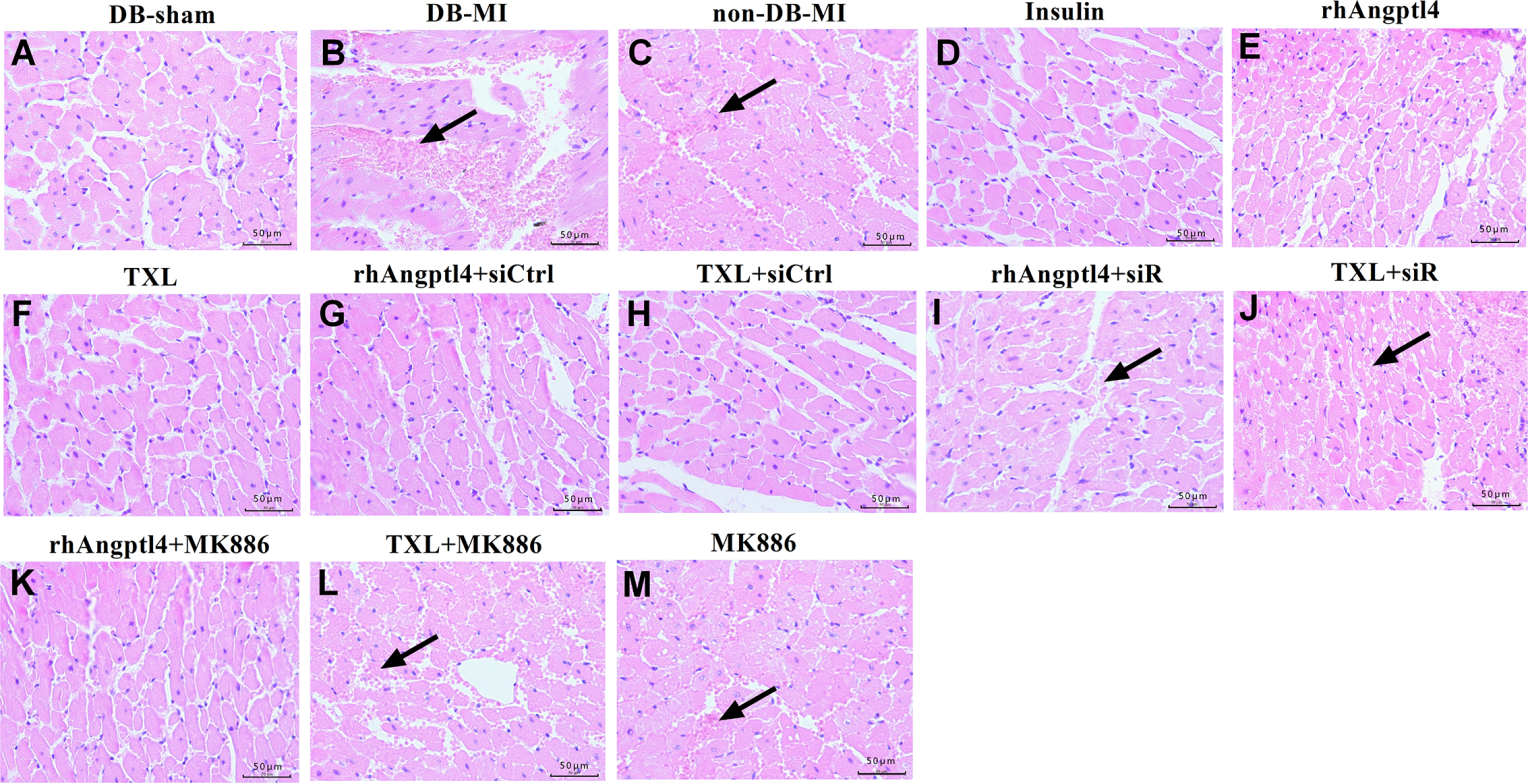
Comparation of parameters of myocardial injury, blood glucose and endothelial barrier integrity in reperfused rats. Area of necrosis, levels of CK activity and cTnI, blood glucose levels, microvascular permeability levels, myocardial focal bleeding score and endothelial cell apoptosis rate was compared among different groups. (A-C) Quantification of myocardial injury and necrosis; (D) Blood glucose levels; (E-G) Parameters of endothelial barrier integrity. Compared with the DB-sham group, *P<0.05, **P<0.01; Compared with the DB-MI group, ^†^*P<*0.05, ^††^*P<*0.01; Compared with the TXL group, ^‡^*P<*0.05, ^‡‡^*P<*0.01; Compared with the rhAngptl4+siR group, ^§§^*P<*0.01; Compared with the Insulin group, ^¶¶^*P<*0.01. N = 8 for each group. Abbreviations as in Fig 1.

As was shown in **Figs 1** and **2A–2C, S1 Fig** and **S1 Table**, I/R induced myocardial infarction as revealed by histopathological analysis and the levels of CK and cTnI. Although the extents of myocardial ischemia were similar among the animals with I/R injury, a significant increase in myocardial necrosis was found in the hearts of diabetic animals compared to the non-diabetic hearts (*P*<0.01). Interestingly, TXL treatment reduced cardiac necrosis, similar to the animals treated with insulin or rhAngptl4 (*P*<0.01). The TXL inhibitory effect on myocardial necrosis could be compromised by downregulation of Angptl4 with the Angptl4 siRNA (*P*<0.01), suggesting the participation of intrinsic Angptl4 in the TXL protection against I/R injury. The PPAR-α inhibitor MK886 also reversed the effects of TXL, but did not affect the rhAngptl4-reduced myocardial necrosis.

To investigate whether the cardioprotection effect of TXL was related with reduction of blood glucose, blood glucose levels were measured at base line and after 180min of reperfusion **(Fig 2D and S2 Table)**. Blood glucose levels of diabetic rats were significantly higher than non-diabetic controls both at baseline and after 180min of myocardial reperfusion (*P*<0.01). Insulin treatment remarkably decreased the levels of blood glucose of diabetic rats at baseline or after 180min of myocardial reperfusion (*P*<0.01). However, treatment with TXL or rhAngptl4 did not affect blood glucose levels both at both time points (*P*>0.01).

### TXL treatment improves endothelial barrier integrity in reperfused diabetic hearts

**Fig 3:**
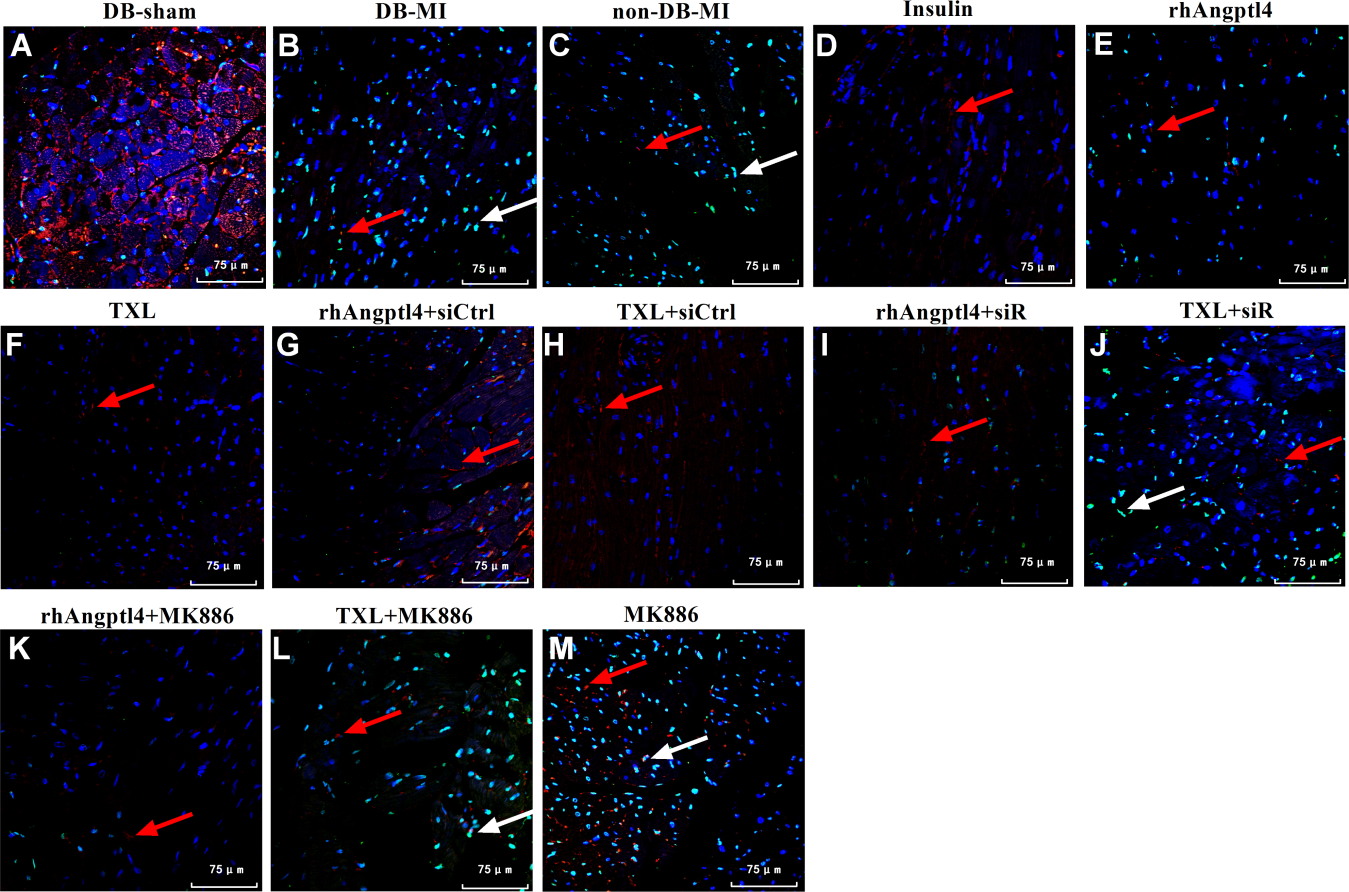
Evaluation of intramyocardiac hemorrhage in the infarcted hearts treated with or without TXL. Sections of the hearts were stained with hematoxylin-eosin (n = 8 in each group). DB-sham group has no obvious extravasation of red blood cells in the interstitial space (A). I/R injury induced apparent extravasation of red blood cells into the interstitial space in both DB-MI (B) and non-DB-MI (C) groups. Treatment with insulin (D), rhAngptl4 (E) or TXL (F) greatly decreased extravasation of red blood cells. Combination with angptl4 siRNA canceled the effects of rhAngptl4 (I) and TXL (J). However, combination with MK886 abolished the effect of TXL (L) but not rhAngptl4 (K). Images were taken under a Leica microscope with 40×objective. Black arrows indicate intra-myocardiac hemorrhage. Abbreviations as in Fig 1.

To evaluate the impact of TXL on microvascular endothelial permeability, FITC-dextran exclusion was quantified (**Fig 2E),** and myocardial focal hemorrhage was scored in the hearts treated with or without TXL **(Figs 2F and 3 and S3 Table)**. I/R injury greatly damaged the integrity of microvascular endothelial barrier, demonstrated by the significant increase of FITC-dextran concentration and focal hemorrhage score in the I/R myocardium of diabetic hearts. Compared with the DB-MI controls, TXL-treated animals showed a remarkable reduction of FITC-dextran concentration and focal hemorrhage score in the reperfused area. Similar effects were found in the diabetic animals treated with insulin or rhAngptl4. The TXL protective effect on endothelial barrier integrity could be partially blocked by both Angptl4 siRNA and MK886 (*P<*0.01). However, MK886 did not affect the rhAngptl4-induced reduction of FITC-dextran concentration and focal hemorrhage. These results suggested that intrinsic Angptl4 participate in TXL protective effect on endothelial barrier function via the PPAR-α pathway.

### TXL treatment decreases apoptosis of endothelial cells in I/R-injured diabetic hearts

**Fig 4:**
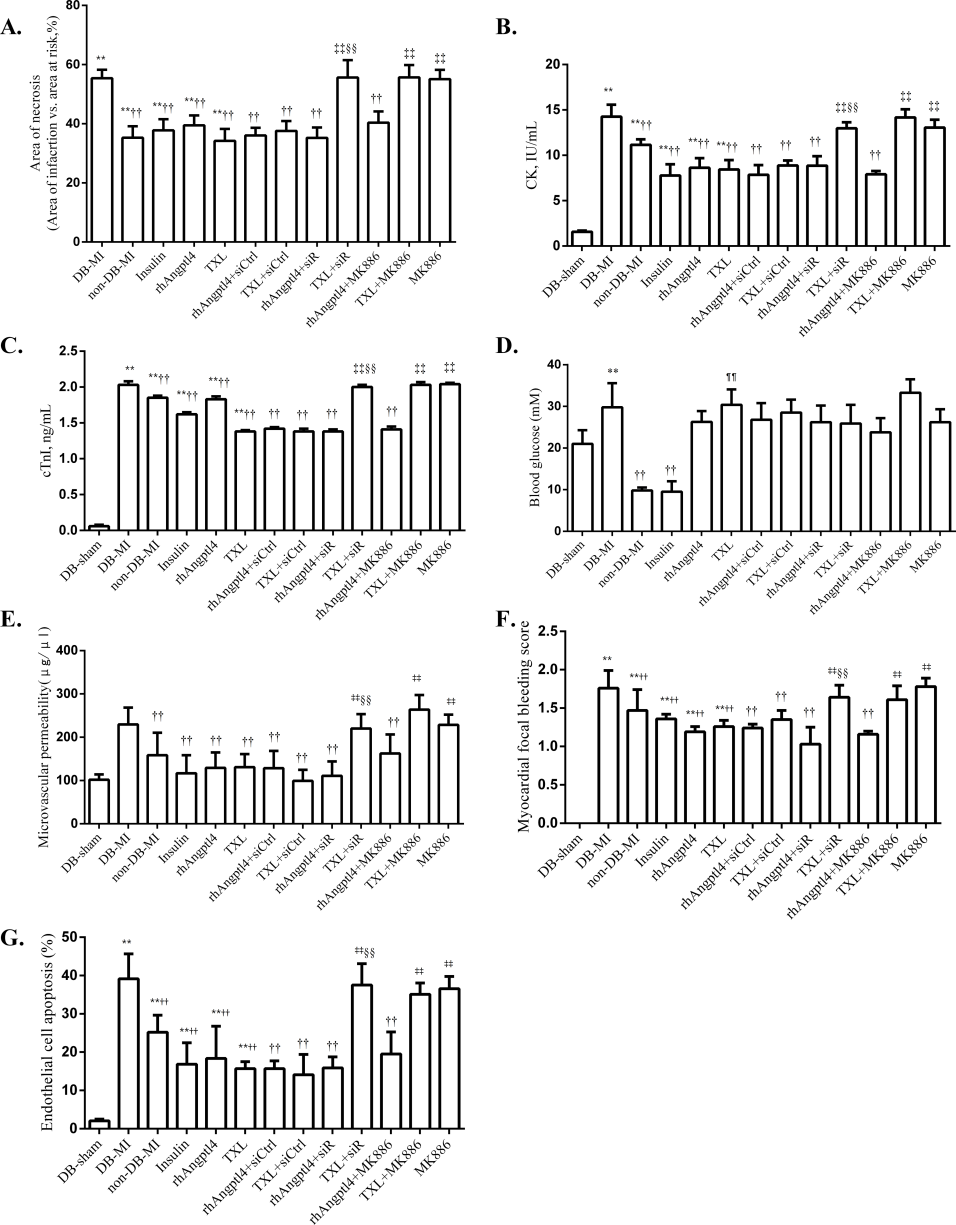
Identification of endothelial cell apoptosis in the I/R-injured hearts treated with or without TXL by confocal microscopy. Endothelial cells were identified by red fluorescence (CD34), total cell number was detected by blue fluorescence (DAPI DNA staining), and apoptosis was detected by green fluorescence (TUNEL). Apoptotic endothelial cells were detected and counted by colocalized red and green (displayed as yellow). I/R injury induced significant ECs apoptosis in both MI control and diabetic MI rats (n = 8 in each group). Treatment with insulin, rhAngptl4 or TXL ameliorated ECs apoptosis compared with the diabetic MI controls. Whereas, co-treatment with Angptl4 siRNA partially blocked the beneficial effect of TXL. Administration of the PPARα inhibitor MK886 also reversed the inhibition effect of TXL on ECs apoptosis, but not reduce ECs apoptosis in the rhAngptl4-treated animals. Red arrows indicate endothelial cells and white arrows show apoptotic cells. Abbreviations as in Fig 1.

The hearts with I/R-injury contained numerous endothelial cells (ECs) bearing the markers and morphology of apoptosis, which were labeled by TUNEL (**Figs 2G and 4 and S3 Table**). Great loss of CD34 staining was found in all images except those from DM-sham group, indicating there were almost no intact ECs in the reperfusion zone of the I/R injured myocardium. I/R injury induced ECs apoptosis in both non-diabetic and diabetic hearts. Treatment with insulin, rhAngptl4 or TXL, led to amelioration of cell apoptosis in comparison to those in the diabetic MI controls. Down-regulation of Angptl4 expression by siRNA interference could partially block the beneficial effect of TXL, but did not have the same effect on rhAngptl4-reduced apoptosis, implying the involvement of intrinsic but not extrinsic Angptl4 in the TXL biological activity. Administration of the PPARα inhibitor MK886 also reversed the function of TXL inhibiting apoptosis. However, interestingly, MK886 could not reduce cell apoptosis in the I/R injured hearts of animals treated with rhAngptl4.

### TXL treatment protects endothelial tight junctions in I/R-injured diabetic hearts

**Fig 5:**
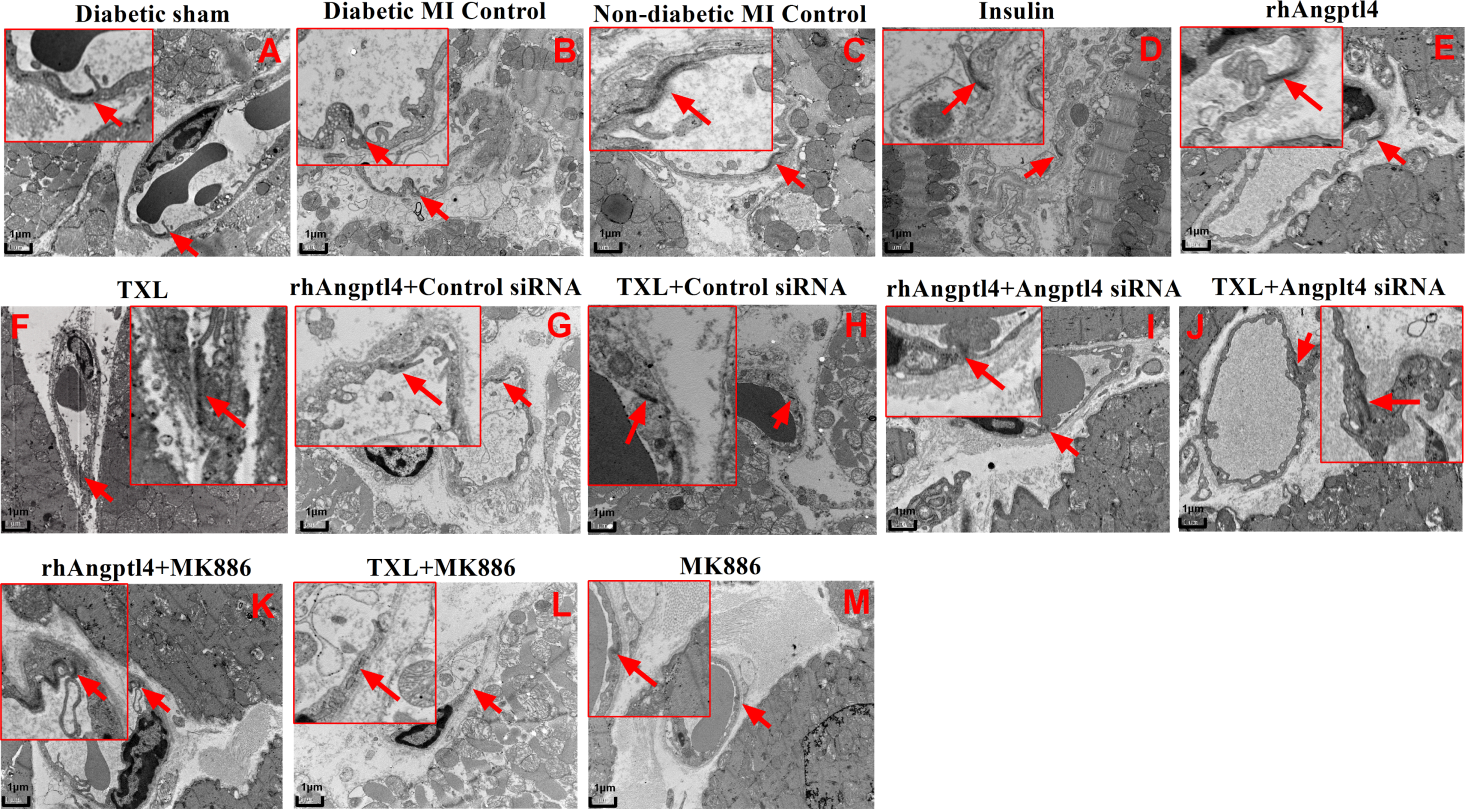
Ultrastructural assessment of the microvascular endothelial tight junction in I/R-injured myocardium. Ultrastructural assessment of endothelial integrity was detected in the infarcted hearts in each group by transmission electron microscopy (×10000 and ×30000) (n = 3 in each group). Arrows indicate tight junctions (TJs). DB-sham group (A) showed a considerable overlap between adjacent ECs. In non-DB-MI (B) and DB-MI (C) groups, no obvious overlap and tight connection were found between adjacent ECs, and ECs appeared swollen with increased lysosomal-like inclusions. Treatment with insulin (D), rhAngptl4 (E) or TXL (F) almost reverted the I/R-induced injury of endothelial tight junctions. Combination with angptl4 siRNA or MK886 neutralized the protective effects of TXL (J, L) on endothelial integrity; whereas combination with angptl4 siRNA (I), but not MK886 (K), suppressed the effect of rhAngptl4. Abbreviations as in Fig 1.

The ultrastructural changes of ECs and their tight junctions (TJs), which plays a key role in maintaining the integrity of endothelial barrier, were visualized using transmission electron microscopy **(Fig 5)**. ECs from sham animals were attached to the basal membrane and appeared elongated and flattened, with a prominent glycocalyx on luminal side and smooth contours. Sham animals showed a considerable overlap between adjacent ECs **(Fig 5A)**. Conversely, ECs from the diabetic hearts with I/R injury appeared swollen with increased lysosomal-like inclusions. The diabetic hearts did show little evidence of the overlap and tight connection between adjacent ECs **(Fig 5B)**. After TXL treatment, however, ECs of the diabetic hearts appeared elongated, attached with smooth contours, and connected well with adjacent ECs **(Fig 5F)**. Similar effects were found in the I/R-injured diabetic hearts treated with insulin **(Fig 5D)** or rhAngptl4 **(Fig 5E)**. Knock-down of Angptl4 with siRNA interference reversed the TXL-mediated recovery of ECs and TJs **(Fig 5J)**. Addition of the PPAR-α inhibitor MK886 also blocked, at least in part, these protective effects of TXL **(Fig 5L)** but not rhAngptl4 **(Fig 5K)**.

### TXL treatment enhances the protein expression of endothelial cell junctions in I/R-injured diabetic hearts

**Fig 6:**
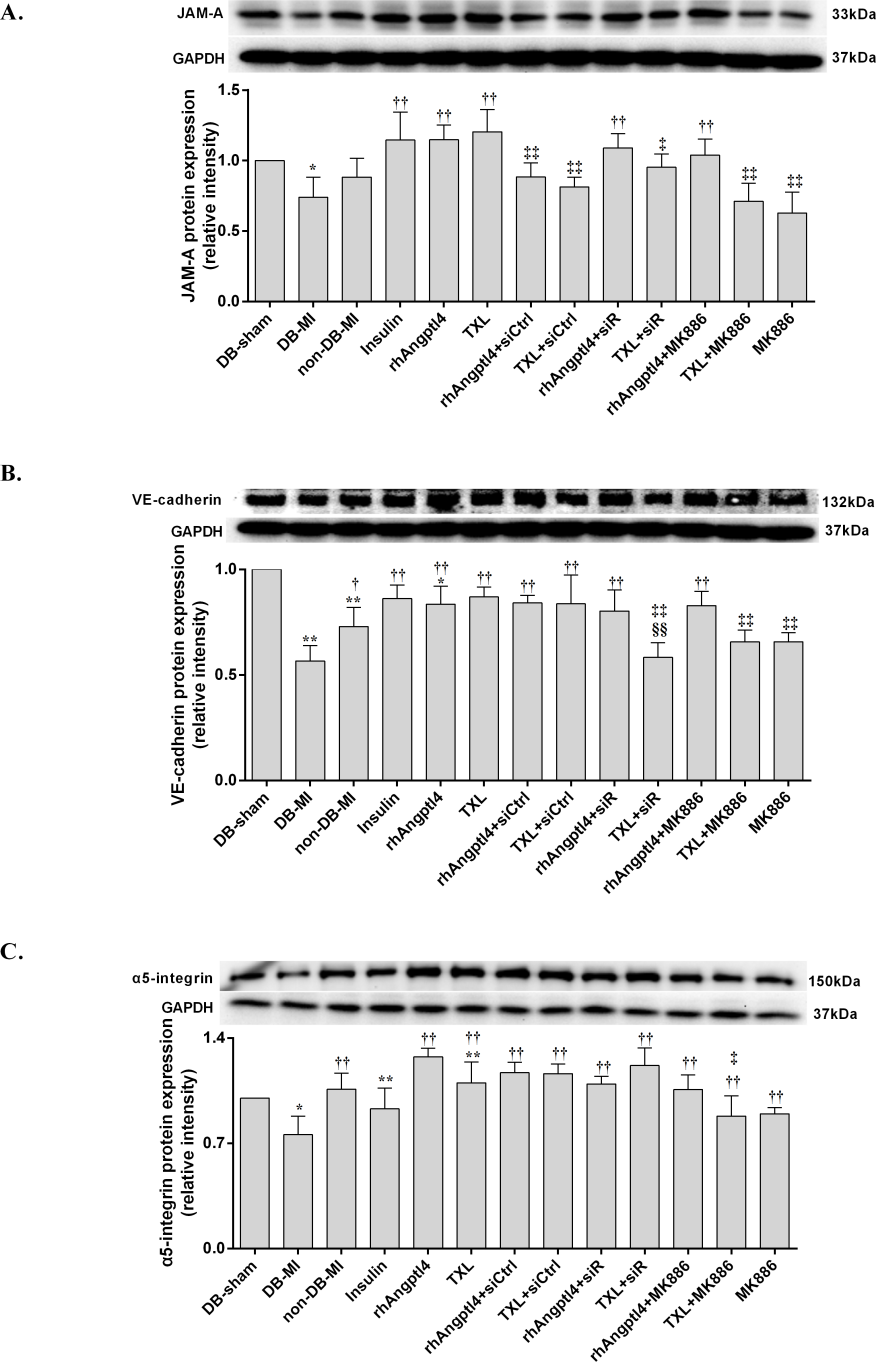
Expression levels of JAM-A, VE-cadherin, and integrin-α5 in the I/R-injured hearts with or without TXL treatment. I/R injury decreased the expression levels of JAM-A (A), VE-cadherin (B), and Integrin-α5 (C). Pre-treatment with insulin, rhAngptl4, or TXL up-regulated expression levels of JAM-A (A), VE-cadherin (B), and Integrin-α5 (C). Addition of Angptl4 siRNA canceled the effects of TXL-induced up-regulation of JAM-A (A) and VE-cadherin (B), but not Integrin-α5 (C). Co-treatment with MK886 abolished the TXL-upregulated expression of JAM-A (A), VE-cadherin (B), and Integrin-α5 (C). Compared with DB-sham group, *P<0.05, ** *P*<0.01; Compared with the DB-MI group, ^†^*P*<0.05, ^††^*P*<0.01; Compared with the TXL group, ^‡^*P*<0.05, ^‡‡^*P*<0.01; Compared with the rhAngptl4+siR group, ^§§^*P*<0.01. Abbreviations as in Fig 1.

To further evaluate the integrity of endothelial barrier in the I/R-injured myocardium, Western blot was performed to detect the levels of endothelial cell tight junction protein JAM-A, adhesion junction protein VE-cadherin, and focal adhesion junction protein integrin-α5 **(Fig 6)**. Significant reductions in the expression levels of JAM-A, VE-cadherin and integrin-α5 were observed in the I/R-injured hearts of diabetic rats. TXL treatment promoted expression of these three proteins. The TXL effect could be mimicked by treatment with rhAngptl4 or insulin, but no significant change in the expression of integrin-α5 was found in the insulin-treated animals. Furthermore, Angptl4 siRNA could inhibit TXL-induced up-regulation of JAM-A and VE-cadherin, but not integrin-α5. Addition of the PPAR-α inhibitor MK886 partially blocked TXL-induced up-regulation of JAM-A, VE-cadherin and integrin-α5, but did not interfere with the rhAngptl4-induced up-regulation of these three proteins.

### TXL treatment stimulates Angptl4 expression in reperfused diabetic hearts via the PPAR-α pathway

**Fig 7:**
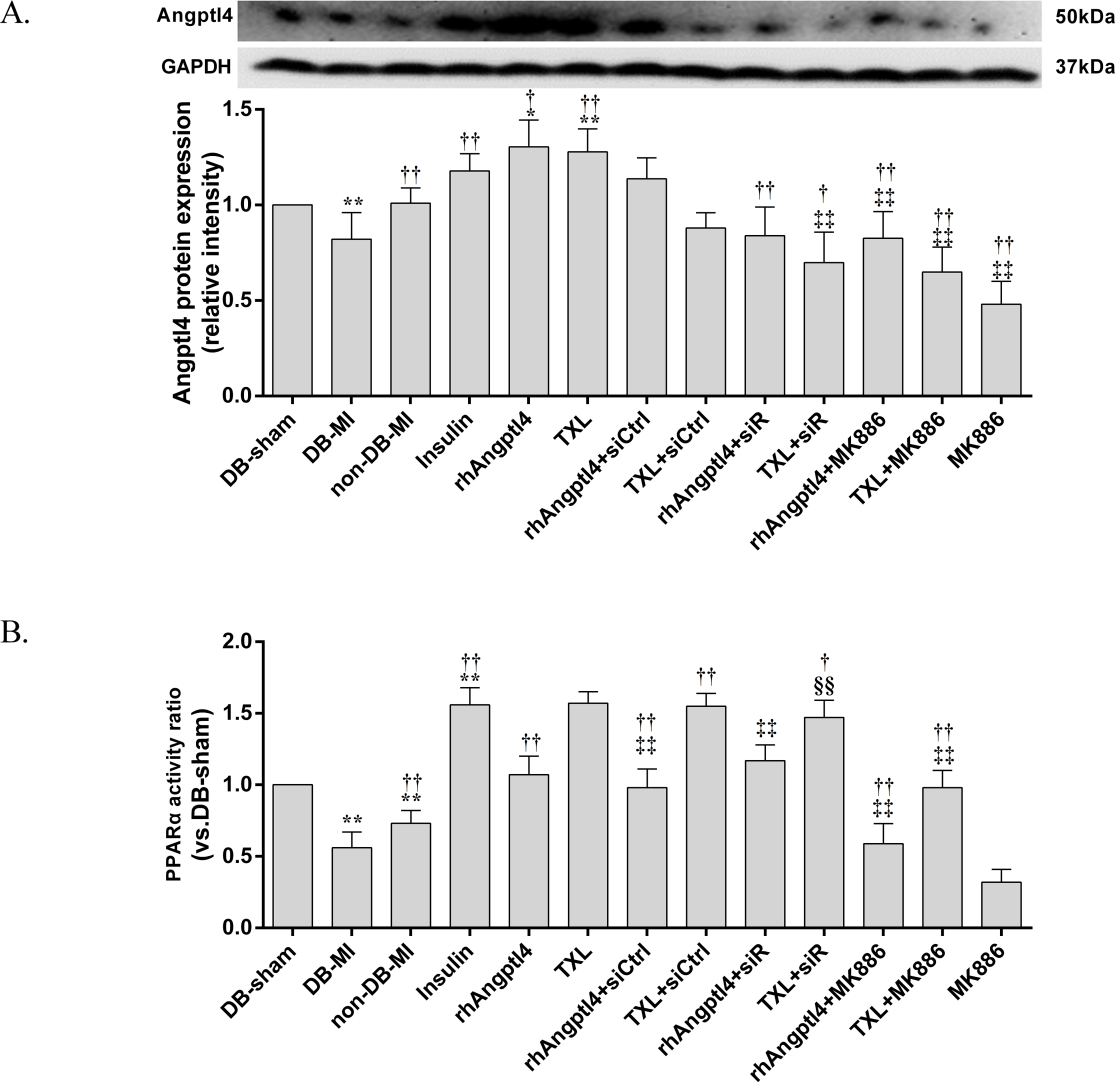
Expression levels of Angptl4 and analysis of PPAR-α activity in the I/R-injured diabetic hearts treated with or without TXL in the presence or absence of signal regulators. (A) Angptl4 expression was decreased by I/R injury, and pre-treatment with insulin, rhAngptl4, or TXL reverted this effect. Whereas, Angptl4 siRNA and MK886 abolished the TXL-induced upregulation of Angptl4. (B) I/R injury decreased the PPAR-α activity in the I/R-injured myocardium, and even worse in diabetic hearts. Pre-treatment of insulin, rhAngptl4 and TXL increased the PPAR-α activity. Addition of MK886 but not Angptl4 siRNA abolished the TXL-stimulated PPAR-α activation. Compared with the DB-sham group, *P<0.05, **P<0.01; Compared with the DB-MI group, ^†^*P*<0.05, ^††^*P*<0.01; Compared with the TXL group, ^‡^*P*<0.05, ^‡‡^*P*<0.01; Compared with the rhAngptl4+siR group, ^§§^*P*<0.01. Abbreviations as in Fig 1.

To explore whether Angptl4 serves as an executant in TXL enhancing the endothelial barrier integrity, the levels of Angptl4 protein was detected in the I/R-injured diabetic hearts by Western blot **(Fig 7A)**. Significant reduction of Angptl4 expression was observed in the I/R-injured hearts of diabetic rats. TXL treatment promoted Angptl4 expression, but not when co-treated with control siRNA. Treatment with rhAngptl4 or insulin enhanced Angptl4 protein levels in the I/R-injured diabetic hearts as well. However, both knock-down of Angptl4 with siRNA interference and inhibition of PPAR-α with MK886 could inhibit TXL-induced up-regulation of Angptl4, suggesting that TXL can modulate the expression of Angptl4 through the PPAR-α pathway.

### TXL treatment elevates PPAR-α activity in diabetic hearts during I/R injury

PPAR-α activity was analyzed to explore whether PPAR-α serves as a commutator in TXL repairing the endothelial barrier integrity **(Fig 7B)**. PPAR-α activity was dramatically decreased in the I/R-injured diabetic hearts. TXL treatment augmented PPAR-α activity, and insulin either. However, rhAngptl4 did not significantly affect the PPAR-α activity in the I/R-injured myocardium of diabetic rats. Furthermore, TXL-induced up-regulation of PPAR-α activity was blocked by addition of MK886 but did not by Angptl4 siRNA, suggesting that TXL-induced activation of PPAR-α is independent of Angptl4, and PPAR-α is the upstream of Angptl4.

## Discussion

In this study, we demonstrated that: 1) TXL can alleviate myocardial I/R injury by protecting endothelial cells and endothelial barrier integrity even in the diabetic rats, in which the microvascular endothelium has been injured or damaged; 2) by preserving the integrity of endothelial barrier function and structure, Angptl4 plays a pivotal role in the effects of TXL against myocardial I/R injury in diabetic rats, partially under regulation of the PPAR-α pathway.

Our study found that diabetic rats is more sensitive to I/R injury than non-diabetic rats, demonstrated by the increased infarct size and cardiac enzymes, consistent with previous studies [19–21]. This is because that diabetes mellitus increases myocardial susceptibility to I/R injury [22]. During diabetes mellitus, O-linked-N-acetylglucosamine (O-GlcNAc) modification of acetaldehyde dehydrogenase 2 (ALDH2), an important cardioprotective enzyme [23], is enhanced, interleukin (IL)-33 is downregulated [19], PKC-βII [19] and xanthine oxidase [22] are activated, thioredoxin-1 is nitratively inactivated [21], thioredoxin-interacting protein (Txnip) [20] and NADPH oxidase [22] are upregulated, nitric oxide synthase is uncoupled [22], and antioxidant capacities is impaired [22], resulting in exaggeration of oxidative stress, inflammation, mitochondrial fission [24] and apoptosis, and subsequently amplification of myocardial I/R injury [19–24].

The present study confirmed that TXL greatly reduced myocardial I/R injury in the diabetic rats, and this effect was independent of blood glucose level. The cardioprotective effects of TXL might be explained by its effects of anti-inflammation [4,5,8,25], anti-oxidative stress [4,5,26], anti-apoptosis [4,5,8], and protection of endothelial barrier integrity [3–6,8]. Although the exact chemical constituents of TXL is not clear, certain chemical compounds in TXL have been demonstrated to contribute to its cardioprotective effects. Ginsenosides, the major active ingredients in Radix ginseng, have antioxidant, anti-inflammatory, anti-apoptotic and immunostimulant properties [27]. Total glucosides of paeony extracted from Radix paeoniae rubra has been revealed to be antioxidant and anti-inflammatory, by inhibiting the secretion of inflammatory factors like intracellular adhesion molecule-1 (ICAM-1), IL-1, IL-6 and TNF-α [28,29], and by balancing secretion of IL-2/IL-4 and IL-10/IL-17 [30]. Betulinic acid from Semen Ziziphi Spinosae possesses the effects of anti-oxidization by inhibiting reactive oxygen species (ROS) production and increasing the activities of SOD and CAT [31], anti-apoptosis by upregulating Bcl-2 and downregulating Bax and caspase-3 via the phosphatidylinositol-4,5-bisphosphate 3-kinase (PI3K)/protein kinase B (Akt) pathway [32], and anti-inflammation by decreasing the levels of IL-6, IL-1β, and TNF-α through the 5'-AMP-activated protein kinase (AMPK)/ nuclear factor (NF)-κB/ transcription factor Nrf2 pathway [31]. Melatonin from Periostracum cicadae, and scolopendra and Semen Ziziphi Spinosae, has the ability of anti-oxidization by scavenging free radicals and ROS, activating antioxidant defense enzymes, and increasing NO bioavailability [26,33,34], anti-inflammation by reducing the expression of P-selectin and ICAM, suppressing NFκB translocation into the nucleus [33], and decreasing endoplasmic reticulum stress [35], anti-apoptosis by up-regulating Bcl-xl and eNOS and down-regulating Bax, nNOS, and iNOS [36], mitochondria-protection by inhibiting the mitochondrial permeability transition pore (mPTP), activating uncoupling proteins (UCPs), reducing mitochondrial fission and elevating their fusion, promoting mitophagy, and improving homeostasis of mitochondria [34], and these effects are probably mediated by the Akt pathway [37].

For diabetes patients receiving reperfusion therapy, insulin is the preferred anti-diabetic drug, as it not only lowers the level of blood glucose but also decreases myocardial I/R injury by reducing myocardial apoptosis [36,38]. In our present study, we as well demonstrated the microvascular-protection effect of insulin in I/R-injured diabetic hearts. The mechanism of insulin attenuates myocardial I/R injury involves improving energy metabolism via increasing glucose uptake [39], and lowering circulating free fatty acids [36]. Besides, studies have demonstrated that insulin maintained the perfusion of microvasculature in a nitric oxide (NO)–dependent manner [40], enhanced the expression of VE-cadherin and β-catenin in human fetoplacental vessels from pregnancies with diabetes [41], protected endothelial barrier function by suppressing endothelial contractile machinery and strengthening cell-cell adhesions through PI3K/Akt- and NO/cGMP-induced Rac1 activation [42]. Furthermore, the microvascular-protection of insulin precedes the induction of glucose uptake, indicating that the cardioprotective effect of insulin is primary but not the consequence of changes in cellular metabolism [40].

In this study, we further confirmed that TXL protected endothelial barrier integrity in reperfused diabetic hearts as effective as insulin. Certain chemical compounds in TXL possess the protective effects on endothelial barrier integrity. Ginsenoside Rb1, the major ingredient of TXL, protects endothelial barrier function by inhibiting VE-cadherin phosphorylation and ZO-1 degradation through the suppression of NF-κB and Src activation [43]. Melatonin, contained in Periostracum cicadae, and scolopendra and Semen Ziziphi Spinosae, reduces neuronal injury and blood-brain barrier (BBB) permeability in an eNOS-dependent manner [37]. Furthermore, Hirudo, Buthus martensii Karsch scorpion [44] and steleophaga plancyi [45] possess anticoagulant or antithrombotic effects, which may also exert protective effects during myocardial I/R injury of diabetes. However, additional studies are needed to detect the detailed mechanism(s) of TXL against myocardial I/R injury following the protection of endothelial barrier integrity, and to determine which compounds from these herbs are responsible for the observed effects presented here. Besides, we noticed that the differences in the hemorrhage scores, albeit significant, are very small. An explanation for this phenomenon is that, in this 45-min ischemia/3-h reperfusion diabetic rat model, the endothelial barrier damage is too small to allow for great leakage of red blood cells but large enough for FITC-dextran (70 kDa) extravasation.

Our study confirmed that, by enhancement of intercellular junctions, Angptl4 played a pivotal role in TXL protecting diabetic heart from I/R injury. It has been demonstrated that lower serum levels of Angptl4 is independently associated with no-reflow after successful PCI in ST-elevation myocardial infarction (STEMI) patients [46]. It was reported that exogenous Angptl4 protected endothelial barrier integrity by inhibiting VEGF-driven dissociation of the VEGFR2/VE-cadherin complex, reduced the size of myocardial infarct and no-reflow in mice and rabbits, but had no direct effect on cardiomyocytes in vitro in hypoxia, suggesting that Angptl4 may protect the heart by acting on endothelial cells, without changing the vitality of cardiomyocytes [9]. However, in tumor models of endothelial cells and mice, Angptl4 caused endothelial barrier damage by integrin signaling and disruption of intercellular VE-cadherin and claudin-5 clusters [47]. These studies suggest that Angptl4 functions in a tissue-dependent fashion.

Present study revealed that TXL protected diabetic heart from I/R injury via the PPAR-α pathway. PPAR-α is highly expressed in the heart, liver, kidney, intestine, and brown adipose tissue, all of which are characterized by an elevated rate of fatty acid catabolism [48]. PPAR-α regulates the cardioprotection effect of fenofibrate + metformin against I/R injury in AMI rats of diabetes, through mechanisms of anti-inflammation and anti-oxidization via the PI3K/Akt/eNOS/NO pathway [49]. Recently, Ginsenosides is confirmed that can prevent ethanol-induced hepatocyte steatosis in vitro by inhibition of oxidative stress and improvement of mitochondrial function through the PPAR-α pathway [50]. Therefore, Ginsenosides may partially contribute to the TXL-induction of PPAR-α pathway.

Further study is needed to clarify the exact compounds of TXL that stimulates the expression of Angptl4. As TXL are complex mixtures of several ingredients, the effects exerted by TXL are believed to be synergistic interactions between the different ingredients and need to be further detected in future studies.

## Conclusions

In a manner similar to insulin and rhAngptl4, TXL could protect the hearts of diabetic rats against I/R injury by maintaining the integrity of endothelial barrier function and structure. Under the regulation of PPAR-α pathway, Angptl4 might serve as the pivotal effector in TXL protecting the I/R-injured diabetic hearts.

## Supporting information

S1 Fig Quantification of area of necrosis. Compared with Diabetic MI Control (DB-MI) group, **P<0.01; Compared with TXL group, ^▲▲^*P<*0.01. Abbreviations as in Fig 1. Data are presented as mean ± SD, n = 8.
(TIF)

S1 Table Quantification of myocardial injury and necrosis. Compared with the DB-sham group, **P<0.01; Compared with the DB-MI group, ^††^*P<*0.01; Compared with the TXL group, ^‡‡^*P<*0.01; Compared with the rhAngptl4+siR group, ^§§^*P<*0.01. Abbreviations as in Fig 1. CK = creatine kinase, AN = area of necrosis. Data are presented as mean ± SD, n = 8.
(DOCX)

S2 Table Comparison of blood glucose levels between groups. Compared with the DB-sham group, **P<0.01; Compared with the DB-MI group, ^††^*P<*0.01; Compared with the Insulin group, ^¶¶^*P<*0.01. Abbreviations as in Fig 1. Data are presented as mean ± SD, n = 8.
(DOCX)

S3 Table Parameters of endothelial barrier integrity in reperfused diabetic hearts. Compared with the DB-sham group, *P<0.05, **P<0.01; Compared with the DB-MI group, ^†^*P<*0.05, ^††^*P<*0.01; Compared with the TXL group, ^‡^*P<*0.05, ^‡‡^*P<*0.01; Compared with the rhAngptl4+siR group, ^§§^*P<*0.01. Abbreviations as in Fig 1. Data are presented as mean ± SD, n = 8.
(DOCX)
