# Supplementary figures and images for: Tongxinluo attenuates reperfusion injury in diabetic hearts by angiopoietin-like 4-mediated protection of endothelial barrier integrity via PPAR-α pathway
Source: PLoS One. 2018 Jun 18;13(6):e0198403. doi: 10.1371/journal.pone.0198403 (PMC6005559; doi:10.1371/journal.pone.0198403)

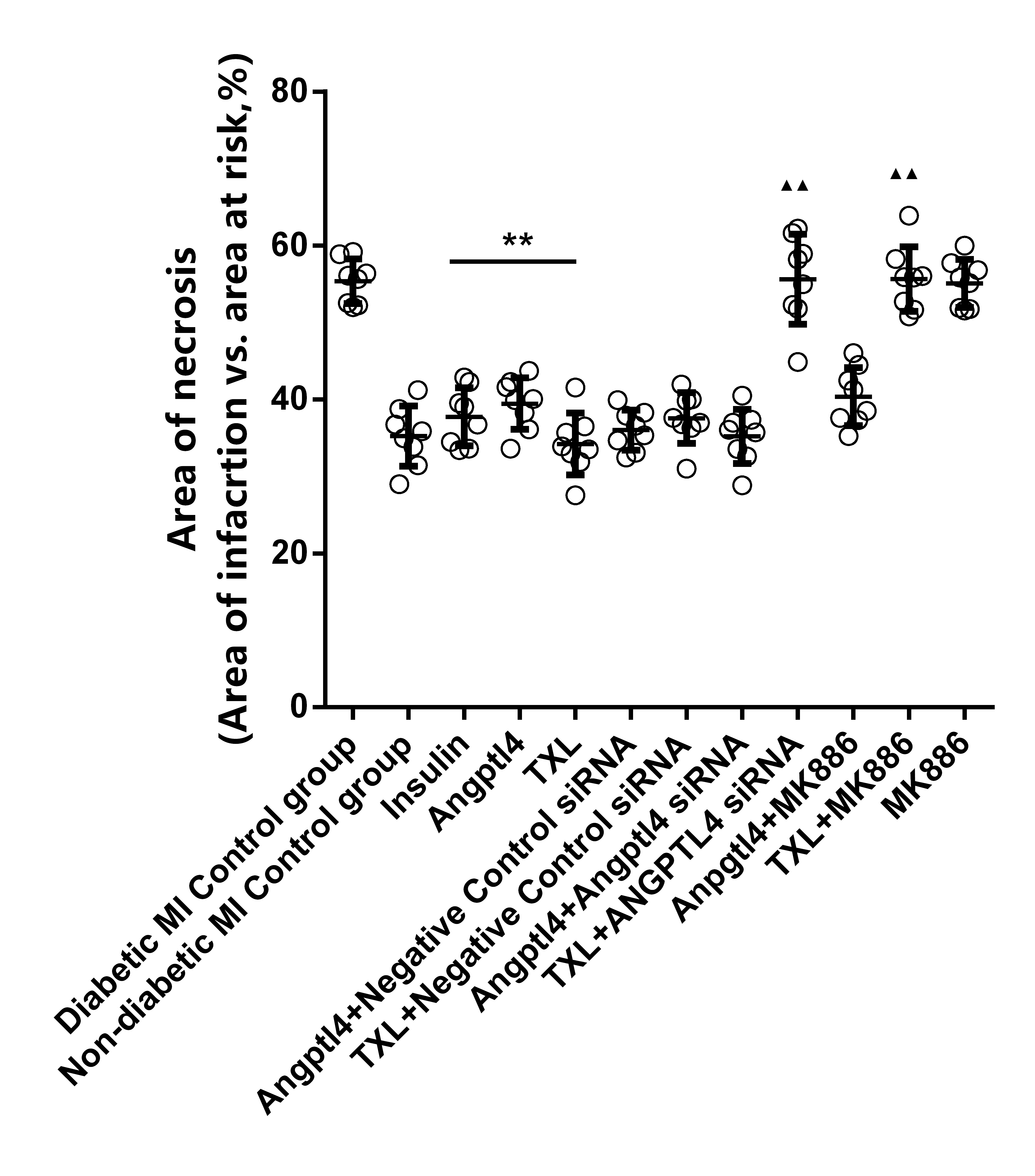

Supplement: S1 Fig — Compared with Diabetic MI Control (DB-MI) group, **P<0.01; Compared with TXL group, ▲▲P<0.01. Abbreviations as in Fig 1. Data are presented as mean ± SD, n = 8. (TIF) [file pone.0198403.s001.tif]
