# Supplementary material for: Tongxinluo attenuates reperfusion injury in diabetic hearts by angiopoietin-like 4-mediated protection of endothelial barrier integrity via PPAR-α pathway
Source: PLoS One. 2018 Jun 18;13(6):e0198403. doi: 10.1371/journal.pone.0198403 (PMC6005559; doi:10.1371/journal.pone.0198403)
Supplement: S1 Table — Compared with the DB-sham group, **P<0.01; Compared with the DB-MI group, ††P<0.01; Compared with the TXL group, ‡‡P<0.01; Compared with the rhAngptl4+siR group, §§P<0.01. Abbreviations as in Fig 1. CK = creatine kinase, AN = area of necrosis. Data are presented as mean ± SD, n = 8. (DOCX) [file pone.0198403.s002.docx]

**S1 Table. Quantification of myocardial injury and necrosis**

|  | **Baseline** | | **180 min after reperfusion** | | |
| --- | --- | --- | --- | --- | --- |
|  | **CK, IU/mL** | **cTnI, ng/mL** | **CK, IU/mL** | **cTnI, ng/mL** | **AN,%** |
| DB-sham | 0.86±0.05 | 0.02±0.01 | 1.58±0.13 | 0.06±0.02 | 0±0 |
| DB-MI | 0.86±0.06 | 0.02±0.01 | 14.25±1.32^**^ | 2.03±0.05^**^ | 55.39±2.88^**^ |
| non-DB-MI | 0.84±0.04 | 0.04±0.01 | 11.16±0.60^**††^ | 1.85±0.03^**††^ | 35.24±3.88^**††^ |
| Insulin | 0.88±0.10 | 0.02±0.01 | 7.77±1.24^**††^ | 1.62±0.03^**††^ | 37.76±3.77^**††^ |
| rhAngptl4 | 0.83±0.07 | 0.02±0.01 | 8.63±1.06^**††^ | 1.83±0.04^**††^ | 39.45±3.34^**††^ |
| TXL | 0.86±0.09 | 0.02±0.01 | 8.45±1.02^**††^ | 1.38±0.02^**††^ | 34.21±4.02^**††^ |
| rhAngptl4+siCtrl | 0.84±0.08 | 0.03±0.01 | 7.86±1.06^††^ | 1.42±0.02^††^ | 36.05±2.60^††^ |
| TXL+siCtrl | 0.78±0.04 | 0.03±0.01 | 8.87±0.55^††^ | 1.38±0.04^††^ | 37.58±3.31^††^ |
| rhAngptl4+siR | 0.78±0.04 | 0.06±0.02 | 8.86±1.05^††^ | 1.38±0.03^††^ | 35.23±3.51^††^ |
| TXL+siR | 0.79±0.04 | 0.04±0.01 | 12.98±0.67^‡‡§§^ | 2.00±0.03^‡‡§§^ | 55.63±5.84^‡‡§§^ |
| rhAngptl4+MK886 | 0.82±0.05 | 0.02±0.01 | 7.90±0.37^††^ | 1.41±0.04^††^ | 40.38±3.79^††^ |
| TXL+MK886 | 0.79±0.08 | 0.04±0.02 | 14.17±0.90^‡‡^ | 2.03±0.04^‡‡^ | 55.66±4.20^‡‡^ |
| MK886 | 0.78±0.02 | 0.04±0.02 | 13.05±0.88^‡‡^ | 2.04±0.02^‡‡^ | 55.10±3.11^‡‡^ |

Compared with the DB-sham group, ^**^*P<*0.01; Compared with the DB-MI group, ^††^*P<*0.01; Compared with the TXL group, ^‡‡^*P<*0.01; Compared with the rhAngptl4+siR group, ^§§^*P<*0.01. Abbreviations as in Fig 1. CK=creatine kinase, AN=area of necrosis. Data are presented as ± SE, n=8**.**
