# Supplementary material for: Tongxinluo attenuates reperfusion injury in diabetic hearts by angiopoietin-like 4-mediated protection of endothelial barrier integrity via PPAR-α pathway
Source: PLoS One. 2018 Jun 18;13(6):e0198403. doi: 10.1371/journal.pone.0198403 (PMC6005559; doi:10.1371/journal.pone.0198403)
Supplement: S2 Table — Compared with the DB-sham group, **P<0.01; Compared with the DB-MI group, ††P<0.01; Compared with the Insulin group, ¶¶P<0.01. Abbreviations as in Fig 1. Data are presented as mean ± SD, n = 8. (DOCX) [file pone.0198403.s003.docx]

**S2 Table. Comparison of blood glucose levels between groups**

|  | | **Blood glucose (mM)** | | |
| --- | --- | --- | --- | --- |
|  | **Baseline** | | **180 min after reperfusion** |
| DB-sham | 21.7±4.1 | | 21.0±3.3 | |
| DB-MI | 22.3±2.4 | | 29.8±5.8^**^ | |
| non-DB-MI | 5.1±1.0^††^ | | 9.8±0.7^††^ | |
| Insulin | 11.4±2.3^††^ | | 9.5±2.5^††^ | |
| rhAngptl4 | 21.0±2.5 | | 26.3±2.6 | |
| TXL | 25.1±3.3^¶¶^ | | 30.4±3.7^¶¶^ | |
| rhAngptl4+siCtrl | 20.1±4.3 | | 26.8±4.0 | |
| TXL+siCtrl | 21.5±3.4 | | 28.5±3.1 | |
| rhAngptl4+siR | 22.1±2.5 | | 26.2±4.0 | |
| TXL+siR | 22.8±2.6 | | 25.9±4.5 | |
| rhAngptl4+MK886 | 21.4±4.2 | | 23.8±3.4 | |
| TXL+MK886 | 21.1±2.2 | | 33.3±3.2 | |
| MK886 | 22.8±3.2 | | 26.2±3.1 | |

Compared with the DB-sham group, ^**^*P<*0.01; Compared with the DB-MI group, ^††^*P<*0.01; Compared with the Insulin group, ^¶¶^*P<*0.01. Abbreviations as in Fig 1. Data are presented as ± SD, n=8**.**
