# Supplementary material for: Tongxinluo attenuates reperfusion injury in diabetic hearts by angiopoietin-like 4-mediated protection of endothelial barrier integrity via PPAR-α pathway
Source: PLoS One. 2018 Jun 18;13(6):e0198403. doi: 10.1371/journal.pone.0198403 (PMC6005559; doi:10.1371/journal.pone.0198403)
Supplement: S3 Table — Compared with the DB-sham group, *P<0.05, **P<0.01; Compared with the DB-MI group, †P<0.05, ††P<0.01; Compared with the TXL group, ‡P<0.05, ‡‡P<0.01; Compared with the rhAngptl4+siR group, §§P<0.01. Abbreviations as in Fig 1. Data are presented as mean ± SD, n = 8. (DOCX) [file pone.0198403.s004.docx]

**S3 Table. Parameters of endothelial barrier integrity in reperfused diabetic hearts**

|  | | **Microvascular permeability**  **(μg/μl)** | | **Myocardial focal bleeding score** | **Endothelial cell apoptosis (%)** | |
| --- | --- | --- | --- | --- | --- | --- |
| DB-sham | 101.70±12.32 | | 0.00±0.00 | | | 2.07±0.47 |
| DB-MI | 229.60±39.01 | | 1.76±0.23^**^ | | | 39.14±6.52^**^ |
| non-DB-MI | 158.40±52.01^††^ | | 1.47±0.27^**††^ | | | 25.18±4.45^**††^ |
| Insulin | 116.90±41.45^††^ | | 1.36±0.06^**††^ | | | 16.86±5.58^**††^ |
| rhAngptl4 | 129.20±35.69^††^ | | 1.19±0.07^**††^ | | | 18.35±8.43^**††^ |
| TXL | 130.80±30.22^††^ | | 1.26±0.08^**††^ | | | 15.67±1.86^**††^ |
| rhAngptl4+siCtrl | 128.50±40.03^††^ | | 1.24±0.05^††^ | | | 15.70±2.02^††^ |
| TXL+siCtrl | 99.31±25.60^††^ | | 1.35±0.12^††^ | | | 14.11±5.27^††^ |
| rhAngptl4+siR | 110.90±33.22^††^ | | 1.03±0.22^††^ | | | 15.91±2.87^††^ |
| TXL+siR | 220.20±33.46^‡‡§§^ | | 1.64±0.16^‡‡§§^ | | | 37.53±5.58^‡‡§§^ |
| rhAngptl4+MK886 | 162.50±44.08^††^ | | 1.16±0.04^††^ | | | 19.50±5.78^††^ |
| TXL+MK886 | 263.90±33.44^‡‡^ | | 1.61±0.18^‡‡^ | | | 35.10±2.95^‡‡^ |
| MK886 | 228.60±23.61^‡‡^ | | 1.78±0.11^‡‡^ | | | 36.58±3.18^‡‡^ |

Compared with the DB-sham group, ^*^*P<*0.05, ^**^*P<*0.01; Compared with the DB-MI group, ^†^*P<*0.05, ^††^*P<*0.01; Compared with the TXL group, ^‡^*P<*0.05, ^‡‡^*P<*0.01; Compared with the rhAngptl4+siR group, ^§§^*P<*0.01. Abbreviations as in Fig 1. Data are presented as ± SD, n=8**.**
